# New Leading-Edge Reinforcement Design of Aircraft Wing to Withstand Bird Collision

**DOI:** 10.3390/biomimetics11050305

**Published:** 2026-04-29

**Authors:** Suppasin Ngamlikitlert, Minsung Kim, Suwin Sleesongsom

**Affiliations:** Department of Aeronautical Engineering, International Academy of Aviation Industry, King Mongkut’s Institute of Technology Ladkrabang, 1 Chalongkrung Rd., Ladkrabang, Bangkok 10520, Thailand

**Keywords:** aircraft wing structure, bird strike, honeycomb material, SPH, optimization

## Abstract

Bird strikes are a key threat to aircraft wing leading edges. This investigation evaluates a honeycomb block reinforcement concept to improve bird strike resistance while maintaining structural efficiency. A validated simulation was developed using an explicit dynamic finite element approach, in which the bird was modeled as a soft body using smoothed particle hydrodynamics, and the wing leading edge was represented with a honeycomb block reinforcement concept. A design of experiments based on McKay Latin hypercube sampling was applied to comprehensively examine the effects of the geometric parameters on the maximum von Mises stress and maximum deformation. Response surface regression models were then constructed to approximate the impact responses and analyze the model correctness. These models were subsequently integrated into a constrained optimization methodology using sequential quadratic programming and population-based integrated learning to minimize deformation while limiting stress below the material yield threshold. The optimized honeycomb and skin configuration demonstrated a noticeable optimization of the maximum deformation within the yield stress limit compared with the baseline design. The results confirm that the proposed honeycomb block reinforcement concept, combined with a regression-based optimization strategy, constitutes a practical, computationally effective approach to improving bird strike resistance and provides a feasible design option for future impact-resistant wing leading-edge designs.

## 1. Introduction

Bird strikes pose a serious threat to aviation safety, with approximately 310,000 wildlife strikes occurring from January 1990 to 2023 [1], particularly during low-altitude operations [2]. The first recorded bird strike occurred on 7 September 1905 in Ohio, when Orville Wright’s aircraft struck and killed a bird [3]. Since then, the scale of the risk has grown alongside the rapid expansion of global air traffic, which is projected to reach around 51 million aircraft by 2030 [3]. Statistics indicate that bird strikes usually occur at low altitude, and these accidents have resulted in 499 human fatalities and the destruction of 361 aircraft worldwide [1,2]. As engines and wings play a key role in generating the basic aerodynamic forces as they generate thrust and lift, respectively, these two components are the main components that are usually hit [2].

These events involve high-speed impacts, transferring a critical amount of kinetic energy to the aircraft structure; such damage can create serious material deformation, structural damage, and catastrophic failure [4], which can cause serious safety hazards, an increase in maintenance costs, and operational delays, leading to major financial losses [5]. Moreover, bird collisions rarely cause catastrophic damage, but they can still result in significant losses to the aviation industry [6]. A bird strike is a rapid process that often occurs in a few milliseconds, so the material under the bird collision may experience intermediate-to-high strain rates, leading to nonlinear high-speed deformation during impact [4,7]. Furthermore, the takeoff speed is fast enough for the bird to become a soft body, as its body is mostly water, and at high speed, birds experience considerable distortion and behave like fluid [8]. Due to the expense and complexity of physical testing, finite element analysis (FEA) has become an important tool for bird strike simulations. Because of its ability to capture significant deformations and fluid-like behavior during high-velocity impacts, smoothed particle hydrodynamics (SPH) has become the most popular meshless method among numerical techniques. SPH is commonly preferred for modeling bird strike events because it is more durable and better correlates with experimental data than conventional mesh-based techniques [9].

Srinivasan (2025) [10] compared the Al 8090 alloy with the traditional Al 2014 alloy in a bird strike event and found that Al 8090 was better in terms of the energy absorption and strain rate performance, while the density was lower. Ericsson (2012) [11] numerically assessed a new leading-edge design using carbon fiber-reinforced plastic (CFRP). Although the result failed the standard, an increasing number of CFRP layers reduced the in-plane spread of delamination. Long (2020) [12] highlighted that increasing the proportion of ±45° piles improved the ability to absorb energy from the carbon fiber–epoxy composite material, as the damage dissipation energy under shear loading was relatively large compared with other directions. Ubels (2003) [13] aimed to determine whether the horizontal tail structure with the tensor-skin concept, a tensor pile deployed under high lateral loading, improved the design. The results showed that the tensor-skin concept increases energy absorption at the leading edge. Liu (2017) [14] studied the performance of the triangle reinforcement component on the tail leading edge. The results showed that as the triangle reinforcement helped divert the impact energy from the impact area, the triangle reinforcement improved the anti-bird strike ability. Liu (2023) [15] also studied the triangle support structure, but in the wing. The study found that 1.2 mm of triangle support was preferable, as it reduced the overall weight and prevented a bird from separating, thereby avoiding internal structural damage. Liu (2018) [7] investigated new structural models that can resist bird collisions using SPH and FEA; they found that the sandwich technique was deformed but not penetrated, and whether it was better than the conventional design. The single-plate support deformed more as the plate’s angle decreased. The deformation of the triangle support varied with the gap from the skin. Tezel (2022) [16] compared all designs on the wing leading edge, including the triangle reinforcement structure (TRS), negative Poisson’s ratio (NPR) structure, and honeycomb structure. The results showed that the TRS helped separate the bird more than absorb energy; the knife effect varied with its thickness. The topometric method performed better than the conventional method and the topological TRS due to its maintained rigidity during impact. The NPR still needed improvement, as it did not yet meet the EASA requirement. Arachchige (2023) [17] found that multilayer foam and honeycomb on the skin performed better than the conventional design, with the honeycomb design 85 percent and 45 percent better than the conventional and foam designs, respectively. Moreover, Di Caprio’s (2019) [6] study showed that the honeycomb sandwich structure on the leading edge improved the design to resist bird strikes, and increasing the skin and honeycomb thickness consistently enhanced the crashworthy behavior of the wing leading edge. Chang (2024) [18] also studied the honeycomb sandwich structure and found that it demonstrated excellent energy absorption and impact resistance under dynamic loading. Finally, Tan (2025) [19] studied how auxetic honeycomb material with a negative Poisson’s ratio can improve in terms of absorbing energy and reducing the damage when compared with a conventional design. A new arc-Z-shaped re-entrant honeycomb structure was developed and shockingly reduced the deformation and specific energy absorption (SEA) by 25.33 and 3.56 percent, respectively. Also, the study showed Ti-6Al-4V as a more suitable material for a honeycomb core than stainless steel, with better SEA and less damage than aluminum alloy.

Despite extensive numerical and experimental investigations of bird strikes and impact-resistant aircraft structures, most of the existing studies have focused primarily on material selection and baseline structural design. Only limited research has incorporated systematic optimization approaches to improve bird strike performance. In particular, the combined use of explicit dynamic simulation with response surface-based optimization techniques, such as design of experiments and response surface modeling, remains largely unexplored for wing leading-edge applications. Based on previous findings that honeycomb-based structures show favorable energy absorption characteristics, this study proposes a novel honeycomb leading-edge block reinforcement concept and uses a regression-assisted optimization system to identify an improved design under bird impact loading.

## 2. Materials and Methods

**Figure 1:**
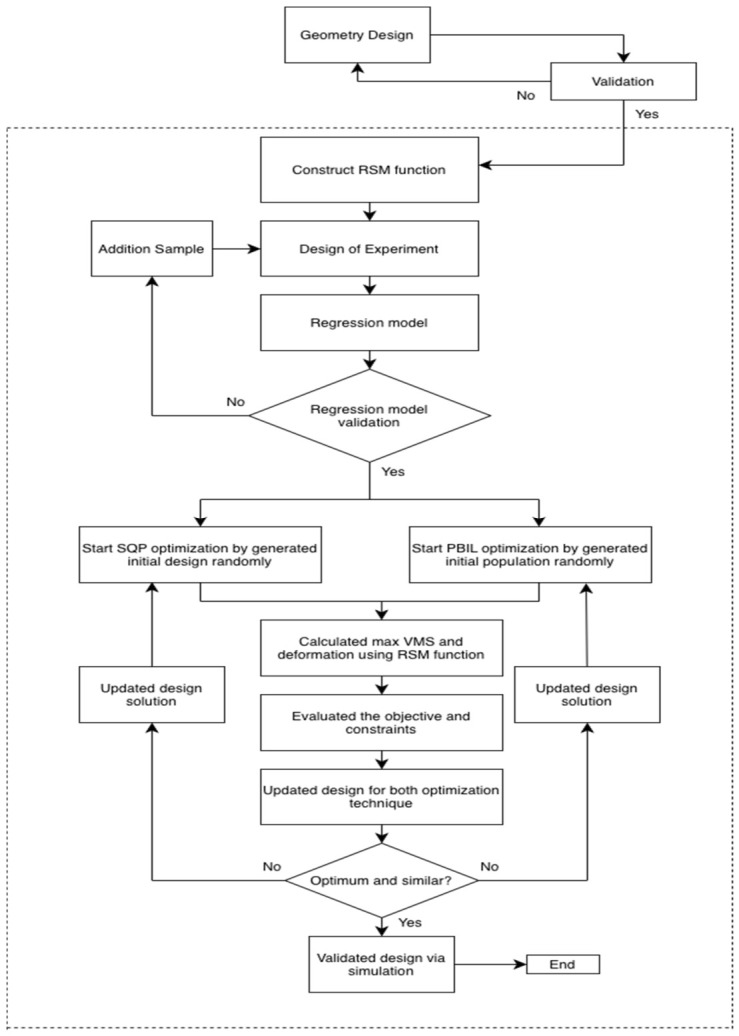
Workflow for aircraft wing leading-edge block concept against bird collision.

This study follows a structured, numerical, and optimization-based workflow to evaluate and improve the bird strike resistance of a novel honeycomb block reinforcement concept (HBRC). The overall methodology, illustrated in the workflow diagram, begins with the geometric design of the leading-edge configuration and its subsequent validation using explicit dynamic simulation. Once the baseline numerical model is verified, a surrogate modeling framework is implemented to lower the computational cost during optimization. A design-of-experiment approach is employed to sample the design space systematically, and the resulting responses are used to construct response surface regression models for predicting the maximum von Mises stress and structural deformation. The accuracy of the regression models is assessed, and additional samples are introduced as required to confirm sufficient predictive performance. Upon successful validation of the surrogate models, two optimization techniques—sequential quadratic programming and population-based incremental learning—are independently applied using randomly generated initial designs. Response surface models are used to evaluate objective functions and constraints in the optimization process. The optimized designs obtained from both techniques are compared for agreement, and the final optimal configuration is confirmed by numerical simulation. The workflow is presented in Figure 1.

### 2.1. Aircraft Wing Structure with Honeycomb Block Reinforcement Concept Design Space

**Figure 2:**
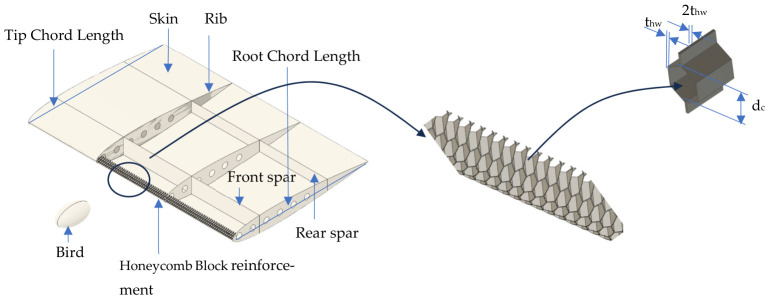
Aircraft wing, bird, and honeycomb models.

This section explains the aircraft wing and bird geometries and bird design, as well as the impact parameters used to analyze bird collisions. The wing model uses a simplified straight-wing design with skin, ribs, front spars, and rear spars. The structure has four ribs joined by front and rear spars. The wing section has a chord length of 1000 mm and a spanwise length of 1500 mm. The front spar is 100 mm from the leading edge. The wing uses an NACA 0012 airfoil profile. The main design variables in this study are the skin thickness, honeycomb cell size, and honeycomb wall thickness. The bird is modeled as a soft, ellipsoidal body with a mass of 1.81 kg (4 pounds). It hits the wing leading edge at an aircraft speed equal to 129 m/s. Other parameters are included in Table 1, and a picture of the wing model is presented in Figure 2.

The honeycomb block reinforcement is modeled as a skin-bonded insert at the leading edge. The insert is attached only to the leading-edge skin and is intentionally not seated on any base member, nor is it mechanically tied to the front spar. This skin-only configuration is selected to manage the bird strike response locally in the leading-edge zone through load spreading and controlled crushing while minimizing direct load transfer into the front spar. This design is consistent with structural design approaches for bird strike resistance that emphasize controlling damage and improving the crashworthiness of leading-edge structures to protect critical internal components [7]. Structurally, the skin-only attachment is conceptually similar to stiffened skin aircraft construction, where reinforcing elements are bonded directly to wing/fuselage skins to enhance local stiffness and stability without requiring a separate base member [20]. The leading edge is treated as the first-impact zone under a bird strike, and its structural concept is evaluated based on how effectively it redistributes impact loads and limits the distribution of damage [6]. Accordingly, the reinforcement effect is expected to appear as increased local through-thickness support to the skin during impact and a broader distribution of deformation and von Mises stress away from the strike point, rather than forcing the front spar to participate in energy dissipation. Design studies on leading-edge structures also indicate that the internal reinforcement arrangement and support strategy are key variables that influence damage localization under bird impact [15,16]. The choice of honeycomb architecture is further motivated by its established impact-energy absorption characteristics, which support its role as a localized energy-absorbing insert [18,19].

#### Design of Experiment

**Figure 3:**
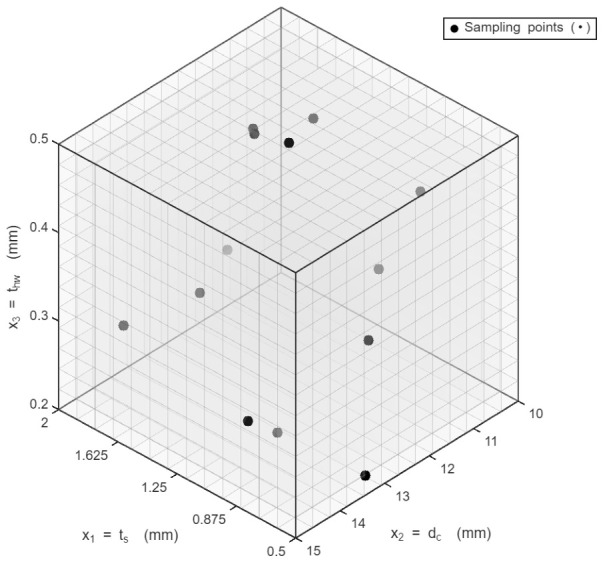
Graphical representation of McKay LHS equal intervals.

Bird strike simulations are highly nonlinear and computationally expensive, making the exhaustive numerical evaluation of all possible design combinations impractical. To address this challenge, design-of-experiment (DoE) techniques have been widely adopted as an efficient statistical framework for exploring the design space while minimizing the number of required simulations [21]. To efficiently sample the design space and generate training data for the regression models, a DoE based on the McKay Latin hypercube sampling (LHS) technique is employed in this study. The McKay LHS method provides uniform coverage of the design space while requiring a relatively small number of simulation runs [22]. There are three variables in this study: x_1_, skin thickness (t_s_); x_2_, honeycomb cell dimension (d_c_); and x_3_, honeycomb wall thickness (t_hw_). In this approach, each design variable is divided into equal-probability intervals. Sampling points are generated so that each interval is sampled exactly once, as shown in Figure 3, ensuring a space-filling distribution of the design points.

In many parametric and optimization studies, some geometric parameters are kept fixed to limit the scope of the investigation and keep computational costs manageable. Using this method, we set the honeycomb insert depth as a constant baseline while varying the internal honeycomb parameters to examine their impact on the impact resistance. This approach enables a fair comparison within a fixed structural envelope and avoids significantly higher simulation costs associated with optimizing both size and configuration simultaneously.

### 2.2. Optimization Objectives and Structural Constraints

The design optimization problem is the minimization of the maximum displacement, subject to the maximum von Mises stress (VMS) constraint and limits of the design variables:

#### Verification of Optimal Design

(1) Minf(x)=max(δ)

Subject to

σ ≤ 167 MPa(2)

x_l_ ≤ x ≤ x_u_ (3)

where x = {x_1_, x_2_, x_3_}^T^, and x_l_ and x_u_ are the upper and lower limits of the design variables, respectively.

### 2.3. Computational Impact Mechanics

This section describes the numerical simulation framework adopted for bird collision analysis. A bird strike event involves high-speed impacts, transferring a critical amount of kinetic energy to the aircraft structure; such damage can create severe material deformation, structural damage, and catastrophic failure [4]. This event is a rapid process, which often occurs in a few milliseconds, so that the material under bird collision may experience an intermediate-to-high strain rate and nonlinear high-speed deformation is caused during the impact [4,7]. Therefore, an explicit dynamic analysis framework is employed in this study using ANSYS AUTODYN (v. 2023).

#### 2.3.1. Model Assumption

The following modeling assumptions are made in this study. First, the bird is modeled as a soft body using water as the substitute material and simulated with the SPH method. Additionally, the wing structure is modeled using shell elements with a simplified straight-wing geometry based on an NACA 0012 airfoil profile. The honeycomb block reinforcement is attached only to the leading-edge skin and is intentionally not connected to the front spar to evaluate the reinforcement concept’s localized energy absorption behavior. The honeycomb insert depth remains constant throughout the study, with only the skin thickness (t_s_), honeycomb cell dimension (d_c_), and honeycomb wall thickness (t_hw_) considered as design variables.

#### 2.3.2. Soft-Body Concept

As the takeoff speed is fast enough for the bird to become a soft body, the bird material is selected to be water, as the bird body composite is mostly water, and at high speed, birds undergo significant distortion and behave like fluid [8]. Hedayati (2022) [23] confirmed that during a bird strike, the bird behaves like a fluid, undergoing extreme distortion, while its body breaks up into debris. Furthermore, Di Caprio (2019) [6], Ubels (2003) [13], and Ericsson (2012) [11] highlighted that a bird during a bird strike impact will behave as fluid. However, a liquid with artificial compressibility is effective only in a limited case, where the velocity is below the physical sound speed [14]. Hence, in this study, the soft-body concept is adopted to replace the complex material behavior of the bird with an equivalent water model, providing a suitable, computationally efficient representation for bird collision simulation.

#### 2.3.3. Smoothed Particle Hydrodynamics (SPH) Method

To accurately capture the large deformation of the fluid-like behavior of the bird during impact, an appropriate numerical formulation is required. In the late 1970s, the SPH (smoothed particle hydrodynamics) numerical method was invented to model the behavior of systems in astrophysics, for which the mesh-based method (Eulerian) was found to be unsuitable due to the lack of a defined boundary. Nowadays, SPH is widely used as the most popular meshless choice because it can predict highly strained motions, such as bird strike simulations, without a computational grid, using a collection of macroscopic particles. It is based on the kernel function, which has the advantage of being applicable in both continuous and discrete settings [14,24]. Heimbs (2011) [25] confirmed that SPH is superior to Lagrangian methods due to mesh distortion issues. Guida (2011) [26] compared SPH with Lagrangian methods and concluded that SPH is superior in terms of robustness and experimental correlation. Still, the Lagrangian method is only acceptable for low and moderate deformations. Matos (2023) [9] compared 4 mesh (Lagrangian, Eulerian, ALE, and CEL) and 3 meshless (SPH/FE to SPH, DEM, and PFEM) methods; the results showed that the mesh method was outperformed by the meshless method due to high soft-body deformation, as accuracy was lost due to numerical error linked with the negative volume, element erosion, and timestep decrement problem. Among all meshless methods, SPH has a standout performance, as this method tracks deformation and history-dependent material behavior very well and is very robust even with large deformation, and it also has the best correlation with experimental data for both the peak force and profile and is excellent for deformation simulation. Based on these advantages, the SPH method is adopted in this study to model the bird during a bird strike incident, providing a reliable, physically appropriate framework for simulating bird collision events involving extreme deformation and high strain rates.

#### 2.3.4. Constitutive Law

The aluminum structure (AL5083H116) is modeled using the Johnson–Cook constitutive law, which accounts for strain hardening, strain rate sensitivity, and thermal softening under high-speed impact loading. The flow stress is expressed as:

(4) $$\sigma =(A+B\varepsilon^{n})\;(1+C\;\ln(\dot{\varepsilon }))\;(1-T_{\mathrm{melt}})$$

where σ is the flow stress; ε is the equivalent plastic strain; $\dot{\varepsilon }$* = $\dot{\varepsilon }$/${\dot{\varepsilon }}_{0}$ is the normalized strain rate with ${\dot{\varepsilon }}_{0}$ = 1/s as the reference strain rate; and T* = (T − T_room_)/(T_melt_ − T_room_) is the homologous temperature. The first bracket (A + Bε^n^) represents the strain-hardening term, describing how the material strengthens as plastic strain accumulates. The second bracket (1 + C ln($\dot{\varepsilon }$)) represents the strain rate term, capturing the increased material strength under high-speed loading conditions characteristic of bird strike events. The third bracket (1 − T_melt_) represents the thermal-softening term, which describes the reduction in flow stress as the material temperature rises during impact. The material constants are A = 167 MPa, B = 596 MPa, n = 0.551, C = 0.001, m = 0.859, and a melting temperature of 619.85 °C.

#### 2.3.5. Treatment of Failure

For the bird material (water), a Tensile Pressure Failure criterion is applied to the SPH water model, allowing the bird particles to separate and disperse upon impact, simulating the fluid-like fragmentation behavior of the bird body during high-velocity collision.

For the aluminum wing structural components, no failure criterion or element erosion was applied in the present study. The simulation focused on the pre-failure deformation and stress response of the wing structure under bird strike loading, consistent with the objective of evaluating the maximum displacement and VMS as the primary structural performance metrics.

#### 2.3.6. Structure Contact

The contact definitions used in this study are designed to accurately model the physical interaction between the bird and the wing structure, as well as the connections among internal structural components. The interaction between the SPH bird particles and the wing structure is described using a Frictionless Body Interaction contact applied to all bodies in ANSYS Explicit Dynamics. This contact method enforces a no-penetration condition between the SPH bird particles and the wing’s shell elements. The internal structural connections between components—including the skin, ribs, front spar, rear spar, and honeycomb block reinforcement—are modeled with bonded contact, ensuring complete and continuous load transfer between all structural parts throughout the simulation. This contact strategy ensures that the structural response captured in the simulation accurately reflects the real interaction between the bird and the wing’s leading edge during a high-speed impact event.

#### 2.3.7. Boundary Condition

The bird is assigned an initial velocity of 129 m/s in the frontal X-direction, with zero velocity in the Y and Z directions, representing a direct frontal impact condition. The wing structure has no fixed positional constraints applied; it remains stationary due to structural inertia during the short impact duration of 5 × 10^−3^ s.

#### 2.3.8. Finite Element Analysis

The structural components of the wing—including the skin, ribs, front spar, and rear spar—were discretized using a mesh element size of 0.025 m with the MultiZone Quad/Tri meshing method, which provides a structured, uniform distribution suitable for capturing the structural response under high-speed impact loading. The honeycomb reinforcement was meshed using a Free Mesh approach due to its complex internal geometry, which required an unstructured mesh to reduce computational costs.

#### 2.3.9. Numerical Model Verification

To verify the numerical model, a bird strike simulation is conducted under benchmark conditions following Timhede (2025) [4]. The bird is modeled as a soft-body ellipsoid using the soft-body concept and SPH, with the wing leading edge impacted at 129 m/s under a direct frontal configuration. The simulation is set up in ANSYS Explicit Dynamics (v. 2023) and solved in AUTODYN. The wing structure and bird are discretized using 6504 elements, while the SPH bird consists of 2082 particles, and the total simulation time is 5 × 10^−3^ s. To verify, the wing structure is made of aluminum in Table 2, with a simplified skin thickness of 1.8 mm, and the bird’s soft-body concept using water properties as shown in Table 3. The verification focuses on the maximum von Mises stress and deformation history.

**Figure 4:**
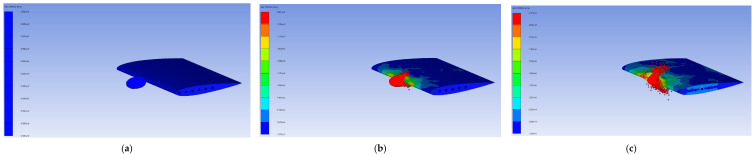

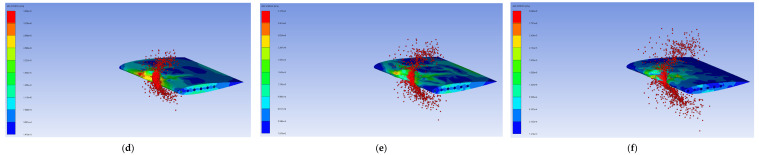
Deformation history of numerical verification model: (**a**) beginning of the event; (**b**) event at 1 ms; (**c**) event at 2 ms; (**d**) event at 3 ms; (**e**) event at 4 ms; (**f**) event at 5 ms.

**Figure 5:**
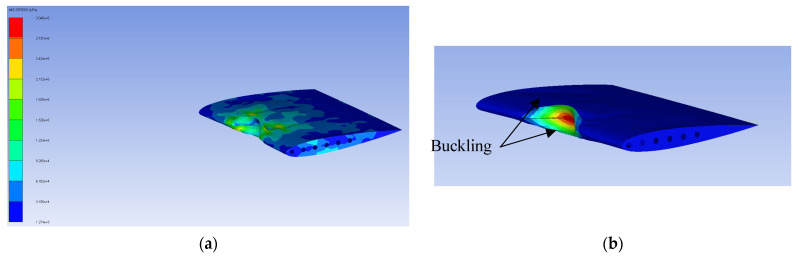
Numerical verification model result: (**a**) VMS distribution result; (**b**) buckling result.

The deformation history of the verification model at each 1 ms interval is presented in Figure 4. The sequence begins with initial skin deformation at the leading edge upon impact, followed by progressive collapse of the leading-edge zone as the bird undergoes fluid-like dispersion, and finally stabilization of the deformed shape after the bird has fully dispersed, a characteristic identical to that of Timhede (2025) [4]. Furthermore, buckling is observed at the upper and lower skins of the leading edge during impact, caused by the compressive load generated by the bird strike. Additionally, the collision effect pulls both the leading edges of the middle ribs together, as shown in Figure 5. This buckling behavior and rib-pulling response are consistent with the results reported by Timhede (2025) [4] and physical experiments by Guida (2011) [26]. The verification model gets a maximum von Mises stress of 304 MPa, whereas the reference study reported a peak stress of 322.4 MPa under a comparable impact condition, as shown in Figure 4. The difference between the present study and that of Timhede (2025) [4] is attributed to the inclusion of front and rear spars, which provide additional load paths and increase the wing’s structural stiffness. Despite this structural difference, the stress concentration region and local buckling behavior of the leading edge remain consistent with the reference paper, confirming that the developed numerical model reliably captures the structural response under a bird strike event.

**Figure 6:**
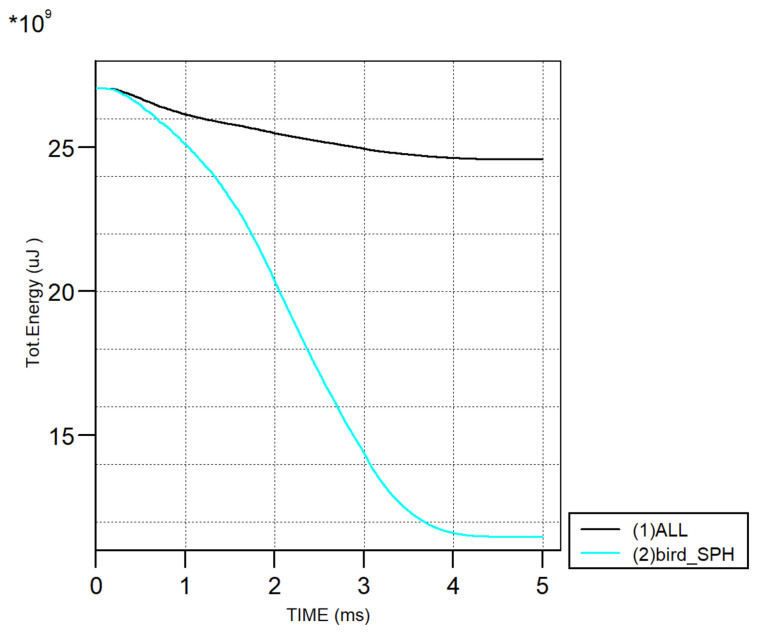
Energy trend of numerical verification model (Total Energy (uJ) vs. TIME (ms) where * = × ).

Moreover, the total energy history shows a rapid reduction during the initial impact phase, corresponding to the transfer of kinetic energy from the bird to the wing structure, followed by stabilization after around 4 ms, as shown in Figure 6, confirming the model’s numerical stability throughout the simulation and verifying these behaviors with Ericsson (2012) [11].

To further substantiate the numerical model’s reliability, the sensitivity of the SPH bird discretization and the structural mesh resolution is examined. Regarding SPH particle refinement, Dede (2015) [27] demonstrated that coarse (10 mm), medium (5 mm), and fine (2 mm) SPH particle resolutions produced similar normalized pressure distributions, with the deformation profile behaving in a near mesh-independent manner. This indicates that the SPH bird response shows limited sensitivity to further particle refinement beyond an adequate resolution. The particle size employed in the present verification model is around 10 mm, which falls within the acceptable range established by this convergence criterion. With respect to structural discretization, the present numerical model employs 6504 elements, compared with 5588 in the reference study by Timhede (2025) [4], resulting in a broadly comparable level of mesh density. The moderate increase in the total element count is attributable to the inclusion of front and rear spars in the present model, which introduces additional structural features necessitating further local mesh refinement. Taken together, these considerations confirm that the chosen discretization parameters are appropriate and that the simulation results are not significantly influenced by mesh or particle resolution effects.

### 2.4. Optimization RSM Employs Regression-Based Technique and Comparative Algorithmic Assessment

The response surface methodology (RSM) describes the relationship between design variables and system responses when direct numerical simulations are computationally expensive. The RSM employs regression-based techniques to approximate complex nonlinear responses using a limited number of simulation results, enabling the efficient prediction of structural behavior across the design space. In structural optimization problems, the RSM is commonly applied after identifying relevant design variables to produce smooth, differentiable response surfaces suitable for subsequent optimization [4]. Previous studies have demonstrated the effectiveness of the RSM at capturing nonlinear effects and interactions among design variables. Uy (2009) [28] discussed the use of response surface models for performance evaluation and optimization, highlighting the importance of second-order polynomial models in representing curvature effects. Furthermore, Box (1960) [29] introduced three-level response surface designs capable of accurately estimating quadratic response functions with substantially fewer experimental runs than full factorial designs. These studies provide the theoretical basis for adopting the quadratic regression-based RSM in the present work.

Moreover, several studies have applied explicit optimization frameworks to the design of aircraft wings and leading-edge structures subjected to bird strike loading. Acar (2025) [30] conducted an optimization-based study of carbon fiber-reinforced polymer (CFRP) structures, treating the laminate thickness and stacking sequence as design variables within an optimal design framework for bird collision scenarios. Wang (2024) [31] investigated a two-step optimization approach for a morphing wing leading edge using a genetic algorithm, in which the optimization sequentially addressed variable skin thickness and a closed-chain mechanism to drive the morphing skin. In addition, Dong (2025) [32] employed topology optimization techniques to determine the optimal material layout and laminate architecture of multilayer composite slats under aerodynamic loading. These studies highlight the use of algorithm-based optimization methods, including laminate optimization, genetic algorithms, and topology optimization, for the design of bird strike-resistant wing structures.

#### 2.4.1. Regression Surface Methodology (RSM)

In this study, a regression-based response surface model is employed to estimate the relationships between the design variables and the structural responses obtained from the bird strike simulation. A second-order polynomial regression model is adopted to capture nonlinear effects and interactions among the design variables. The general form of the response surface function is expressed as

y = β_0_ + β_1_x_1_ + β_2_x_2_ + β_3_x_3_ + β_11_x^2^_1_ + β_22_x^2^_2_ + β_33_x^2^_3_ + β_12_x_1_x_2_ + β_13_x_1_x_3_ + β_23_x_2_x_3_ + ε (5)

where y denotes the predicted structure response; x_1_, x_2_, and x_3_ represent the design variables; β_0_ is the intercept term; β_1_, β_2_, and β_3_ are the linear coefficients; β_11_, β_22_, and β_33_ are the quadratic coefficients; β_12_, β_13_, and β_23_ are the interaction coefficients; and ε represents the residual error of the regression model.

#### 2.4.2. Statistical Validation

The validity and consistency of the regression-based response surface models are evaluated using statistical validation criteria before their application in the optimization process. The coefficient of determination (R^2^) quantifies the proportion of variance in the simulation data explained by the regression model and ranges from 0 to 1. In addition, the adjusted coefficient of determination (R^2^_adj_) accounts for the number of regression parameters and helps reduce the risk of overfitting. A response surface model is considered to have a good fit when the R^2^ is greater than 0.8 and the R^2^_adj_ is greater than 0.7 but less than the R^2^ [4]. These criteria are adopted in the present study as reference guidelines for assessing the adequacy and reliability of the constructed regression models before proceeding with optimization. The RSM is coded in MATLAB software R2024b.

#### 2.4.3. Sequential Quadratic Programming (SQP)

SQP is a gradient-based optimization technique for solving nonlinear constrained optimization problems. The method reformulates the original nonlinear problem into a sequence of quadratic programming (QP) subproblems, in which a quadratic model and the constraints are locally approximated by linear terms about the current design point. By iteratively solving these QP subproblems, the design variables are updated until the convergence criteria for optimality and feasibility are satisfied. Generally, SQP methods have been developed to reliably solve large-scale, constrained, nonlinear optimization problems involving many variables and constraints. These methods require relatively few function evaluations and converge under certain conditions on the problem structure, making SQP well-suited for complex engineering design optimization tasks, including those that employ regression-based surrogate models to represent nonlinear structural responses [33,34]. In this study, SQP uses DoE and the RSM. These models provide smooth approximations of the maximum von Mises stress and displacement responses, enabling efficient optimization without the need for the repeated high computational costs of explicit dynamic simulations. The optimization is performed using the SQP algorithm implemented in MATLAB R2024b, subject to predefined structural constraints and design-variable bounds.

#### 2.4.4. Population-Based Incremental Learning (PBIL)

PBIL is a probabilistic optimization algorithm that combines concepts from genetic algorithms and competitive learning. Instead of evolving an explicit population of candidate solutions, PBIL uses the search space as a probability vector that is iteratively updated toward regions based on the best-performing solutions. This enables the efficient exploration of complex, nonlinear design spaces while maintaining relatively low computational costs. PBIL has been applied to optimization problems involving bird strike-resistant wing structures, where the design space is highly nonlinear and expensive to evaluate through direct numerical simulation. Timhede (2025) [4] employed several optimizers and found that PBIL performs the best in a surrogate-based optimization framework for optimizing aircraft wing leading-edge structures subjected to a bird strike event. In their approach, regression-based response surface models were used to approximate structural responses, and PBIL was applied to search for optimal design variables efficiently within the model domain. In the present study, PBIL was adopted as a global optimization technique to explore the design space defined by the response surface models. The probabilistic search mechanism of PBIL reduces sensitivity to initial conditions. It allows for the effective identification of promising design regions, making it well-suited for optimizing wing leading-edge structures under bird collision loading.

## 3. Results

This chapter presents the numerical results from the bird strike simulations conducted in this study. The results are first reported in the form of a DoE to systematically explore the design space of the honeycomb leading-edge configuration. Based on the DoE results, regression and response surface models are developed and subsequently employed for design optimization using two optimization approaches—SQP and PBIL. The optimized configurations obtained from both optimization methods are then reported and compared. Finally, a conventional leading-edge configuration is evaluated as a reference for the optimized configuration.

### 3.1. Design of Experiment (DoE) Results

**Figure 7:**
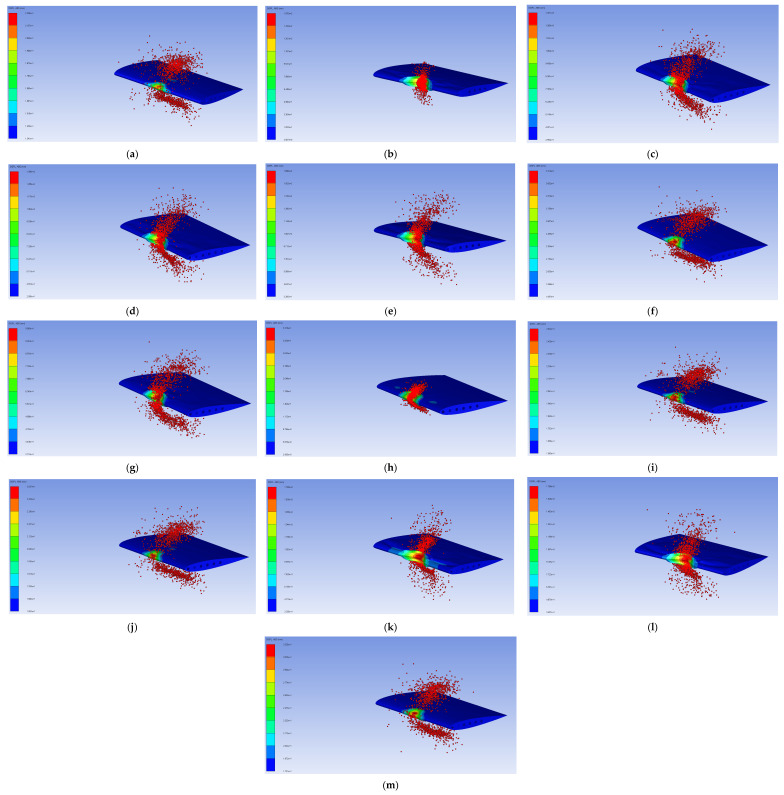
Displacement results from 13 simulations: (**a**–**m**) are runs 1 to 13.

**Figure 8:**
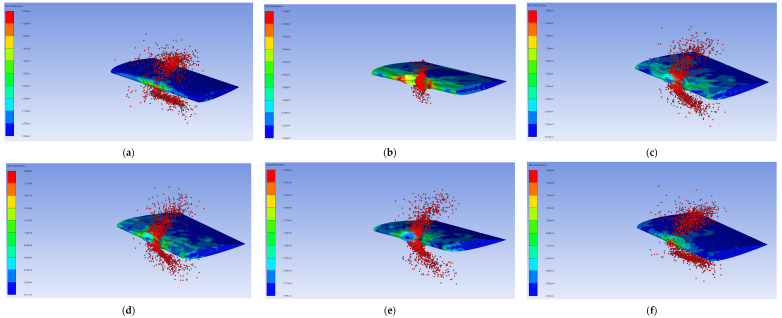

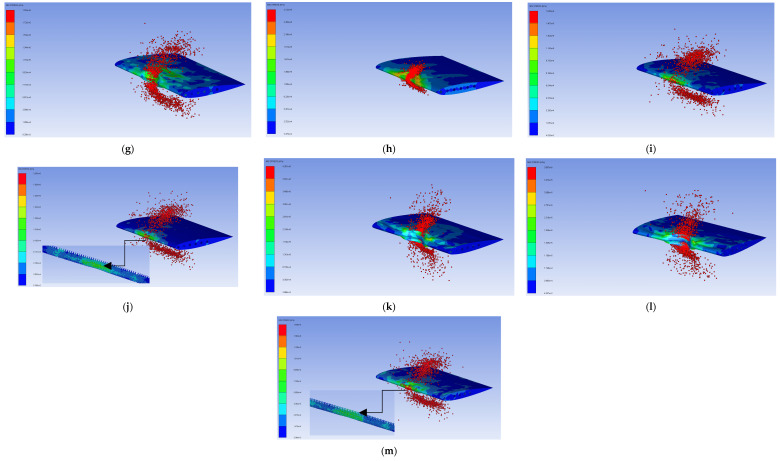
VMS results from 13 simulations: (**a**–**m**) are runs 1 to 13.

Table 4 presents the DoE design matrix employed in this study. A total of 13 simulation runs were generated using a McKay LHS scheme to carefully survey the predefined design space of the honeycomb leading-edge structure. Each run represents a unique dimensional setup defined by a specific combination of three design variables: the skin thickness (x_1_ = t_s_), honeycomb cell dimension (x_2_ = d_c_), and honeycomb wall thickness (x_3_ = t_hw_). These variables were systematically varied within the prescribed bounds described in the Materials and Methods Section to ensure adequate coverage of the design space. For each DoE configuration, a bird strike simulation was conducted under identical conditions. The structural response of the leading edge was evaluated in terms of the maximum displacement (y_1_) and maximum VMS (y_2_), which were extracted from the numerical results and adopted as the primary response quantities. The corresponding deformation and stress contours for each simulation run, along with the associated maximum values, are presented in Table 5, Figure 7, and Figure 8, respectively. The numerical representations provide a detailed review of the impact response across the investigated design space, with each result shown below.

### 3.2. Finite Element Analysis Results

The total number of elements in each DoE run varies from approximately 50,000 to 80,000 elements, depending on the honeycomb cell dimension (d_c_) and wall thickness (t_hw_) specified for each configuration, as the honeycomb geometry changes across runs.

### 3.3. Regression Surface Methodology (RSM) Results

Based on the DoE results, response surface models were constructed for the maximum displacement (y_1_) and maximum VMS (y_2_) using the predefined design variables. These models are used to represent the numerical simulation results within the investigated design space.

Two separates quadratic RSMs were developed in this study. The first model predicts the maximum displacement (y_1_), while the second model predicts the maximum VMS (y_2_). Both response models were formulated as functions of the same three design variables (x_1_, x_2_, and x_3_). The RSM was expressed as a second-order polynomial regression equation (5). The regression coefficients for each response were obtained by least-squares fitting of the DoE data.

The forecasting precision of the developed response surface models was evaluated using the coefficient of determination (R^2^) and the adjusted coefficient of determination (R^2^_adj_). For the maximum displacement response, the model obtained R^2^ = 0.9793 and R^2^_adj_ = 0.9172, while for the maximum VMS response, the model reached R^2^ = 0.9497 and R^2^_adj_ = 0.7988; its adequacies are presented in Table 6 and Table 7. Although the adjusted coefficient of determination (R^2^_adj_) for the VMS model remained within the acceptable range, it was considered relatively low compared with that of the displacement model. Therefore, an additional residual analysis was performed to further examine the model adequacy, as shown in Table 7. The model adequacies of both models are presented in Table 6 and Table 7, which present the residual (e_i_) and R Student (t_i_) values. The residual and R Student of displacement are accepted because the R^2^ and R^2^_adj_ go beyond the acceptable range, but the VMS model needs improvement a bit. The VMS model can be improved by reconsidering the R Student. Based on Table 7 and the VMS model criterion (|t_i_| > 2), which means unusual data, this model can be improved by excluding this data. Run 3 was identified as an outlier in the VMS response and excluded from the final regression. After refitting the models using the remaining 12 runs, the updated model adequacy improved to R^2^ = 0.9872 and R^2^_adj_ = 0.9297 for the VMS model. These values indicate that both response surface models provide satisfactory agreement with the numerical simulation results obtained from the DoE. The regression coefficients are presented in Table 8.

**Figure 9:**
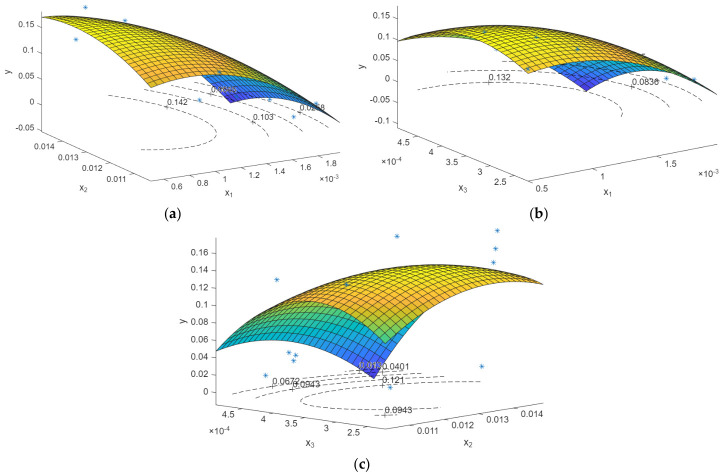
Figure plotting x_1_, x_2_, and x_3_ with maximum displacement (y_1_): (**a**) x_1_ and x_2_ with y_1_; (**b**) x_1_ and x_3_ with y_1_; (**c**) x_2_ and x_3_ with y_1_. The blue asterisks (*) represent the DoE data points, and the color distribution indicates contour level from low (blue) to high (yellow).

**Figure 10:**
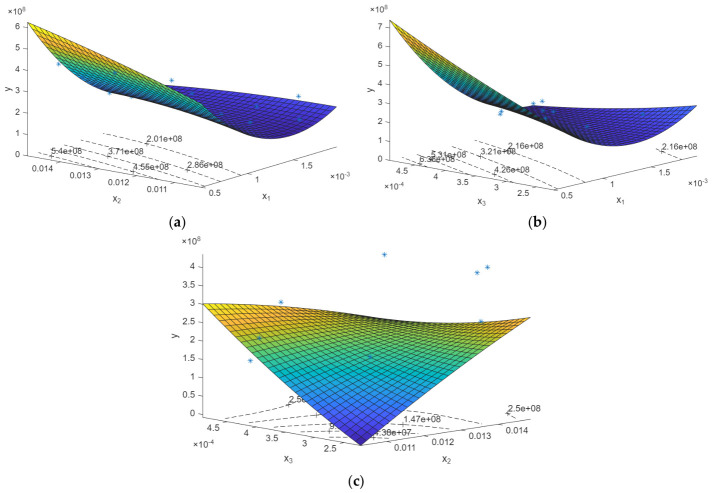
Figure plotting x_1_, x_2_, and x_3_ with maximum VMS (y_2_): (**a**) x_1_ and x_2_ with y_2_; (**b**) x_1_ and x_3_ with y_2_; (**c**) x_2_ and x_3_ with y_2_. The blue asterisks (*) represent the DoE data points, and the color distribution indicates contour level from low (blue) to high (yellow).

To visualize the influence of the design variables on the structural response, response surface plots were generated based on the developed regression models. These plots provide a graphical representation of the predicted response behavior within the investigated design space. For each response quantity, three response surface plots are presented, illustrating the response’s variation as a function of two design variables. In contrast, the third variable is held constant at its mid-range value. The discrete DoE data points are superimposed on the response surfaces to indicate the locations of the simulation runs used to construct the model. In total, six response surface plots are shown, with three plots corresponding to the maximum displacement response presented in Figure 9 and the others corresponding to the maximum VMS response in Figure 10. Together, these plots provide a visual overview of the response surface models developed from the DoE results.

### 3.4. Optimization Result

Based on the RSM developed, an optimization study was conducted to identify improved honeycomb leading-edge configurations for a bird strike. The optimization was carried out using two approaches: SQP and PBIL. Both methods used the same response surface models and design bounds to ensure a consistent basis for comparison.

Table 9 summarizes the optimized geometric parameters obtained using SQP and PBIL. Both methods converged to identical designs inside the prescribed bounds.

The optimized structures obtained using SQP and PBIL are summarized in Table 9. The SQP solution resulted in a maximum deformation of −0.1353 mm. In comparison, the PBIL technique resulted in a maximum deformation of −0.2053 mm. Both are within the VMS constraint, as shown in Table 10.

It should be noted that the displacement values reported in Table 8 represent the predicted structural responses generated by the RSM at the identified optimal design points rather than physically simulated results. These values are the mathematical outputs of the second-order polynomial regression function, which the optimizer internally evaluates during the search process to minimize the objective function. The occurrence of negative predicted displacements is a normal characteristic of RSM-based optimization, and these values should be interpreted solely as internal optimization outputs used to guide the search process and not as representative of the true physical structural response.

**Figure 11:**
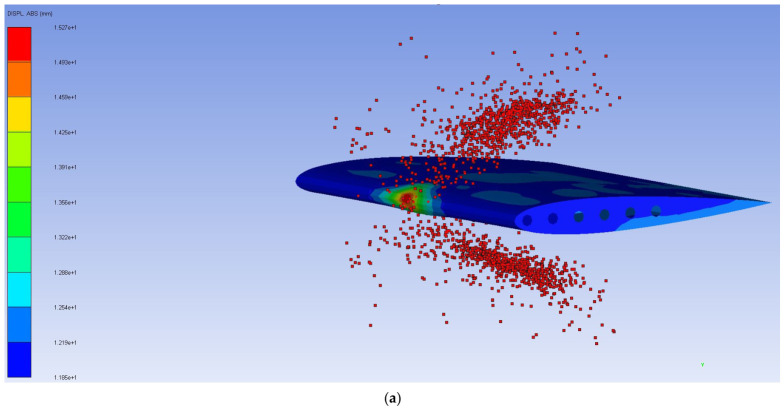

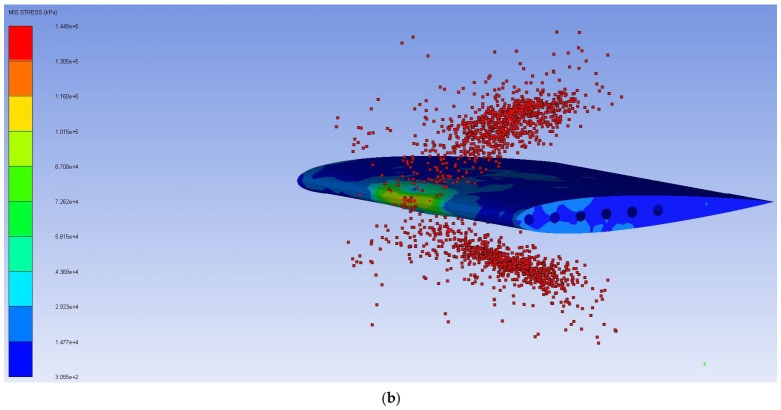
Validation results for optimized design: (**a**) maximum displacement and deformation distribution; (**b**) maximum VMS and VMS distribution.

After validation through simulation, the optimized honeycomb design results are 15 mm and 144.9 MPa for the maximum displacement and maximum VMS, respectively, as shown in Figure 11 and Table 11.

**Figure 12:**
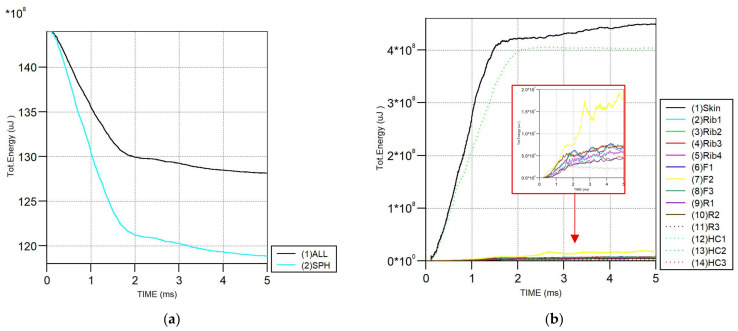
Comparison of total energy of optimized honeycomb model. (1) Left side; (2) middle part; (3) right side; (F) front spar; (R) rear spar; (HC) honeycomb block reinforcement; and (SPH) bird: (**a**) comparison of wing with bird; (**b**) comparison of each wing part.

Figure 12 present the total energy development during the bird strike for the optimized honeycomb models. A rapid reduction in the total energy is observed during the initial impact, showing effective energy transfer from the bird to the wing structure. The component-wise energy distribution shows that the skin absorbs the most significant portion of the impact energy. At the same time, the honeycomb core in the middle region (HC) also contributes significantly to energy absorption after impact, assisting in load redistribution. The ribs and remaining internal components present lower energy levels, confirming their role in structural support rather than primary energy absorption. Overall, the optimized model displays stable, physically consistent energy responses.

### 3.5. Conventional Design

**Figure 13:**
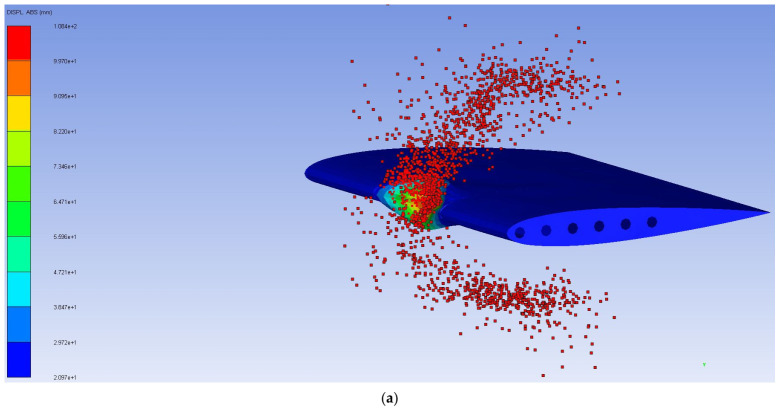

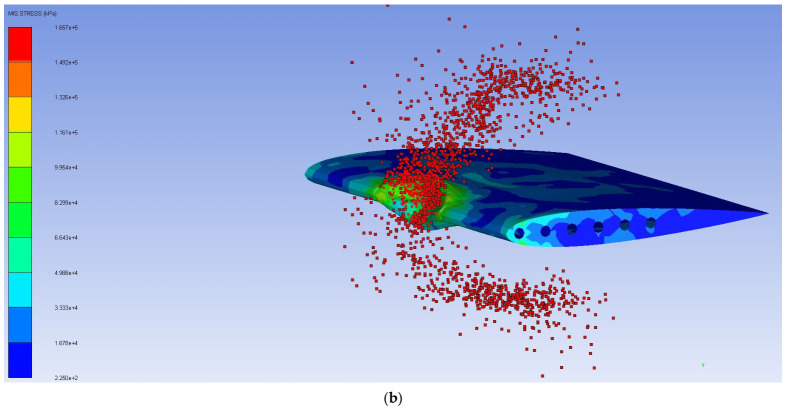
Conventional results: (**a**) maximum deformation and deformation distribution; (**b**) maximum VMS and VMS distribution.

The conventional wing configuration, consisting of a 2 mm aluminum skin without a honeycomb core, exhibits localized deformation at the leading-edge impact region under bird strike loading. The maximum displacement reaches 108.4 mm, while the peak von Mises stress is 165.7 MPa. The stress and deformation responses remain concentrated near the impact zone, indicating that the skin and internal supporting members primarily govern the structural response in the absence of the honeycomb block reinforcement shown in Figure 13. Furthermore, to offer a thorough comparison between the conventional and optimized designs, Table 12 summarizes the structural performances of both configurations. The percentage deformation reduction is nearly 85.91%, while the VMS reduction is 12.55%. Especially for the deformation, the success of the honeycomb reinforcement leading edge against bird strike reduced the deformation close to 85% [17].

**Figure 14:**
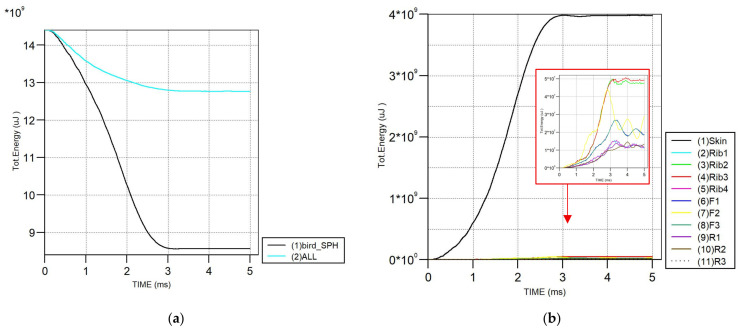
Comparison of total energy of conventional model. (1) Left side; (2) middle part; (3) right side; (F) front spar; and (R) rear spar: (**a**) comparison of wing with bird; (**b**) comparison of each wing part.

The total energy history of the system shows a rapid reduction during the initial impact phase, corresponding to energy transfer from the bird to the wing structure. The energy comparison between the bird and the overall system indicates that a significant portion of the impact energy is dissipated through structural deformation. The component-wise energy distribution further shows that the skin absorbs the dominant share of the impact energy. At the same time, the ribs and internal members exhibit comparatively lower energy levels throughout the simulation. The deformation contours, von Mises stress distribution, and energy evolution for the conventional design are presented in Figure 14.

## 4. Discussion

The results presented in this study provide a comprehensive assessment of the bird strike response of a wing leading-edge structure, encompassing structural deformation, stress distribution, energy absorption, and optimization performance. By combining conventional and honeycomb-reinforced configurations with surrogate-based optimization and complete numerical verification, this study offers insight into both the physical impact mechanisms and effectiveness of the proposed design framework.

In conventional wing configuration, the impact response is mainly governed by the aluminum skin and internal supporting members. The maximum deformation and VMS are highly localized at the leading-edge impact region, indicating that the absence of a honeycomb core limits the structure’s ability to redistribute impact loads. This specific response is also reflected in the energy plotting, where a rapid transfer of kinetic energy from the bird to the wing occurs during the first few milliseconds, followed by stabilization of the total system energy. Component-wise energy analysis shows that the skin absorbs the majority of the impact energy. At the same time, the ribs and internal members contribute comparatively little, confirming their secondary role in reducing energy in the conventional design.

The introduction of the honeycomb block reinforcement substantially changes this response. In the honeycomb block reinforcement concept, the impact energy is no longer predominantly absorbed by the skin alone. Instead, the honeycomb core in the middle region actively participates in energy dissipation, as indicated by its increasing energy contribution after impact. This additional energy absorption mechanism reduces the severity of localized deformation and limits stress concentration at the leading edge. As a result, the structural response becomes more distributed, with improved load transfer across the wing structure. The deformation and stress contours further support this observation. Compared with the conventional design, the honeycomb-reinforced models exhibit broader stress distributions and reduced deformation localization, indicating enhanced structural stiffness and improved impact resistance. Despite this redistribution, no excessive stress concentration is observed in other regions of the wing, and the yield stress of the material nearly exceeds that in the conventional model. However, with the help of the honeycomb block reinforcement concept, the VMS is substantially reduced, demonstrating that the honeycomb block reinforcement effectively improves the impact performance without introducing adverse structural effects.

The energy evolution plots for the optimized configurations also indicate stable and physically consistent behavior. The total energy stays well controlled throughout the simulation, with no abnormal energy growth, confirming numerical consistency. The component-level energy histories reveal consistent trends across different optimized designs, with the skin remaining the dominant energy absorber. At the same time, the honeycomb core provides an additional, meaningful contribution to energy reduction. This combined mechanism clarifies the improved impact response observed in the optimized configurations. The optimization results further demonstrate the effectiveness of the proposed surrogate-based framework. Both the SQP and PBIL algorithms converge to feasible designs that satisfy the stress constraint while decreasing structural deformation. Although the two optimization methods use different search strategies, their optimized solutions exhibit similar deformation trends, stress levels, and energy absorption behaviors when verified through full explicit dynamic simulations. This consistency indicates that the optimization outcome is governed by the structure’s underlying physical response rather than by algorithmic effects. Verification of the optimized designs confirms the reliability of the response surface models. The agreement between the estimated and numerically verified results indicates that the regression models successfully capture the dominant nonlinear relationships among geometric parameters and bird strike response. Consequently, the optimization process yields physically meaningful designs that improve impact resistance while sustaining structural integrity.

Overall, the combined analysis of the deformation, stress, energy dissipation, and optimization outcomes demonstrates the vital role of the honeycomb block reinforcement in enhancing the bird strike resistance of wing leading-edge structures. By enabling additional energy absorption and promoting load redistribution, the honeycomb-reinforced and optimized configurations show clear advantages over the conventional skin-only design. The results confirm that the proposed numerical and optimization framework provides a practical and reliable approach for the design of bird strike-resistant wing structures.

## 5. Conclusions

This study developed and applied a numerical optimization workflow to improve the bird strike resistance of a wing leading edge using a honeycomb block reinforcement concept. An explicit dynamic model was used in ANSYS Explicit Dynamics/AUTODYN, where the bird was modeled as a soft body using SPH, and the wing leading edge included skin, ribs, and spars with a honeycomb block reinforcement inserted as a reinforcement concept.

A benchmark bird strike case was used to validate the numerical setup. Under comparable frontal-impact conditions, where the bird was modeled as an SPH ellipsoid, the developed model reproduced the expected leading-edge stress concentration and deformation. This result supports its suitability for comparative design assessment and optimization studies.

To reduce the computational cost during optimization, a surrogate framework was implemented using a McKay Latin hypercube sampling (LHS) design-of-experiment (DoE) approach. Specifically, the framework used 13 runs over three geometric variables: the skin thickness (t_s_), honeycomb cell dimension (d_c_), and honeycomb wall thickness (t_hw_). Following this, response surface methodology (RSM) regression models were developed to predict the maximum von Mises stress (a measure of material yielding) and maximum deformation. The fitted models achieved strong agreement with the DoE dataset; however, to improve the R^2^_adj_ of the VMS function, cutting the third run out improved it, reporting R^2^ = 0.9793 and R^2^_adj_ = 0.9172 for the displacement function and R^2^ = 0.9872 and R^2^_adj_ = 0.9297 for the VMS function. Together, these results demonstrate that the regression models captured the dominant response trends across the investigated design space.

Two optimizers—SQP and PBIL—were then applied to minimize maximum deformation while constraining von Mises stress below the material yield threshold. Both approaches converged to identical designs within bounds, and the final solutions were verified using full explicit simulations. The validated, optimized designs achieved peak stress levels of 144.9 MPa and corresponding maximum deformation values of 15.27 mm, satisfying the stress constraint and confirming the reliability of the RSM optimization pipeline.

A conventional leading-edge model (2 mm aluminum skin without a honeycomb core) was also evaluated as a reference case. The conventional configuration exhibited localized impact response at the leading edge, with a peak von Mises stress of 165.7 MPa and a maximum deformation of 108.4 mm, indicating that the skin and internal members primarily governed the response, without a dedicated energy-absorbing core.

Overall, the results confirm that the proposed honeycomb block reinforcement concept, which combines a DoE–RSM surrogate and constrained optimization using SQP/PBIL, offers a practical and computationally efficient framework for improving wing leading-edge bird strike performance while keeping stress below the yield limit.

## Figures and Tables

**Table 1:** Wing with honeycomb block reinforcement concept and bird configuration.

No.	Parameters	Values/Shape
1	A section spar length (mm)	1500
2	Root chord length (mm)	1000
3	Tip chord length (mm)	1000
4	Number of ribs	4
5	Rib thickness (mm)	2
6	Distance from leading edge to front spar (mm)	100
7	Honeycomb block reinforcement is the highest (mm)	40
8	Front spar thickness	5
9	Rear spar thickness	6
10	Skin thickness (mm)	0.5–2 (optimization)/1.2 (numerical verification)
11	Honeycomb cell dimension (d_c_, mm)	10–15 (optimization)/N/A (numerical verification)
12	Honeycomb wall thickness (t_hw_, mm)	0.2–0.5 (optimization)/N/A (numerical verification)
13	Aircraft speed (m/s)	129
14	NACA	0012
15	Bird shape	Ellipsoid
16	Bird mass (kg)	1.81

**Table 2:** Wing with honeycomb block reinforcement concept structure material properties.

Aluminum (AL5083H116) Properties	Value	Unit
Young’s modulus (E)	70 × 10^9^	Pa
Yield stress (σ_y_)	167 × 10^6^	Pa
Poisson’s ratio (ν)	0.3	-
Density (ρ)	2700	kg/m^3^

**Table 3:** Bird material properties.

Water 2 Properties	Value	Unit
Mass	1.81	kg
Poisson’s ratio (ν)	-	-
Density (ρ)	1000	kg/m^3^
Grüneisen Coefficient	0.28	-
Parameter C1	1483	m/s
Parameter S1	1.75	-
Parameter S2 (Quadratic)	0	-

**Table 4:** Simulation configurations.

Run	x_1_ = t_s_ (mm)	x_2_ = d_c_ (mm)	x_3_ = t_hw_ (mm)
1	1.97	11.305	0.269
2	0.5	13.434	0.222
3	1.11	10.961	0.32
4	1.03	10.308	0.395
5	0.95	13.806	0.239
6	1.69	11.724	0.446
7	1.34	14.172	0.37
8	1.27	12.414	0.492
9	1.65	10.515	0.424
10	1.82	14.172	0.287
11	0.63	12.894	0.346
12	0.84	14.865	0.295
13	1.53	12.269	0.473

**Table 5:** Results of 13 simulations.

Run	y_1_ (mm)	y_2_ (MPa)
1	22.26	204.3
2	157	413
3	137.7	249.5
4	139.6	235.8
5	168.8	270
6	32.14	180.4
7	99.69	191.6
8	32.16	263.2
9	25.22	162.9
10	25.37	203
11	178	435.7
12	176.8	385.7
13	32.25	144.6

**Table 6:** Model adequacies of maximum displacement (y_1_) function.

Run	y_i_ (mm)	${\hat{y}}_{i}$(mm)	e_i_ (mm)	t_i_
1	22.26	17.2	5.06	2.0680
2	157	160.4	−3.4	−1.1426
3	137.7	144.7	−7	−0.5712
4	139.6	126.2	13. 4	1.9682
5	168.8	165.1	3.7	0.7267
6	32.14	19.6	12.54	0.8896
7	99.69	103.3	−3.61	−0.2661
8	32.16	43.2	−11.04	−1.5207
9	25.22	43.7	−18.48	−3.3973
10	25.37	30.8	−5.43	−1.1881
11	178	181.0	−3	−1.0966
12	176.8	170.7	6.1	0.6530
13	32.25	21.2	11.05	0.6520

**Table 7:** Model adequacies of maximum VMS (y_2_) function.

Run	y_i_ (MPa)	${\hat{y}}_{i}$	e_i_ (MPa)	t_i_
1	204.3	214.09	−9.79	−1.4558
2	413	414.86	−1.86	−0.2222
3	249.5	214.51	34.99	2.1820
4	235.8	259.87	−24.07	−1.2163
5	270	286.40	−16.4	−2.8631
6	180.4	157.10	23.3	0.6934
7	191.6	218.01	−26.41	−1.0504
8	263.2	248.50	14.7	1.5112
9	162.9	164.54	−16.4	−0.0517
10	203	189.49	13.51	1.4478
11	435.7	438.03	−2.33	−0.3113
12	385.7	367.44	18.26	0.9577
13	144.6	176.86	−32.26	−0.9127

**Table 8:** Regression coefficients of equations for y_1_ and y_2_.

Regression Coefficient (β)	y_1_ Model	y_2_ Model
β_0_	−1.56853	−2.35626 × 10^9^
β_1_	3.75254 × 10^2^	7.13601 × 10^10^
β_2_	1.72488 × 10^2^	2.30519 × 10^11^
β_3_	3.00915 × 10^3^	5.5289 × 10^12^
β_11_	−1.474 × 10^4^	−4.16313 × 10^13^
β_22_	−1.73287 × 10^5^	−1.40271 × 10^15^
β_33_	−8.13201 × 10^4^	−3.12866 × 10^14^
β_12_	−9.95885 × 10^4^	2.90353 × 10^14^
β_13_	−5.06535 × 10^3^	−1.88344 × 10^12^
β_23_	−2.92501 × 10^6^	8.67374 × 10^14^

**Table 9:** Geometry parameters obtained by both optimizers.

Optimization Method	x_1_ = t_s_ (mm)	x_2_ = d_c_ (mm)	x_3_ = t_hw_ (mm)
SQP	2	15	0.5
PBIL	2	15	0.5

**Table 10:** Results of both optimization techniques.

Optimization Method	y_1_ (mm)
SQP	−0.1353
PBIL	−0.2053

**Table 11:** Validation results of both optimizers.

Optimization Method	y_1_ (MPa)	y_2_ (mm)
SQP	144.9	15.27
PBIL	144.9	15.27

**Table 12:** Comparison of results.

Design	y_1_ (mm)	y_2_ (MPa)	VMS Constrain Satisfied?
Conventional	108.4	165.7	Yes (nearly unsatisfying)
Optimized Honeycomb	15.27	144.9	Yes

## Data Availability

The data presented in this study are available in article.
